# Dietary Inflammatory Index and Risk of Colorectal Cancer in Japanese Men

**DOI:** 10.3390/nu18020338

**Published:** 2026-01-21

**Authors:** Ayaka Kotemori, Kumiko Kito, Motoki Iwasaki, Taiki Yamaji, James R. Hébert, Junko Ishihara, Manami Inoue, Shoichiro Tsugane, Norie Sawada

**Affiliations:** 1 Department of Food and Life Science, School of Life and Environmental Science, Azabu University, 1-17-71 Fuchinobe, Chuo-ku, Sagamihara 252-5201, Japan; kotemori@azabu-u.ac.jp (A.K.); kitou@azabu-u.ac.jp (K.K.); j-ishihara@azabu-u.ac.jp (J.I.); 2 Division of Cohort Research, National Cancer Center Institute for Cancer Control, 5-1-1 Tsukiji, Chuo-ku, Tokyo 104-0045, Japan; moiwasak@ncc.go.jp (M.I.); mnminoue@ncc.go.jp (M.I.); stsugane@ncc.go.jp (S.T.); 3 Division of Epidemiology, National Cancer Center Institute for Cancer Control, 5-1-1 Tsukiji, Chuo-ku, Tokyo 104-0045, Japan; tyamaji@ncc.go.jp; 4 Department of Epidemiology and Biostatistics, Arnold School of Public Health, University of South Carolina, Columbia, SC 29208, USA; jhebert@mailbox.sc.edu; 5 Cancer Prevention and Control Program, University of South Carolina, Columbia, SC 29208, USA; 6 Department of Nutrition, Connecting Health Innovations LLC, Columbia, SC 29201, USA; 7 Graduate School of Public Health, International University of Health and Welfare, 4-1-26 Akasaka, Minato-ku, Tokyo 107-8402, Japan

**Keywords:** Eastern Asia, inflammation, colorectal cancer, epidemiology, interaction, lifestyle

## Abstract

**Background/Objectives**: Unhealthy lifestyles lead to chronic low-grade inflammation, increasing the risk of colorectal cancer. Few studies in East Asia have examined the association between the dietary inflammation potential and colorectal cancer incidence. Therefore, we aimed to investigate this association further in the Japanese population. **Methods**: This study included 38,807 men aged 45–74 years who participated in the Japan Public Health Center-based prospective study (JPHC Study). The energy-adjusted dietary inflammatory index (E-DII) was derived from a food frequency questionnaire. Hazard ratios (HRs) and 95% confidence intervals (CIs) were estimated using Cox proportional hazards regression models. Differences in risk due to a combination of E-DII and lifestyle were examined using interaction term. **Results**: During 14 years of follow-up, 1415 colorectal cancer cases occurred. A tendency to increased colorectal cancer risk was observed with consumption of pro-inflammatory diets among Japanese men (adjusted HR [95% CI] for the highest quintile: 1.20 [0.99–1.46], *p* trend = 0.08), with a significantly increased risk of colon cancer (HR: 1.28 [1.01–1.63], *p* trend = 0.03). A possible interaction was observed with alcohol consumption (*p* = 0.07), which was statistically significant for proximal colon cancer (HR: 1.14 [1.05–1.25] in drinkers; *p* interaction = 0.01). No significant interactions with other lifestyle factors were found. **Conclusions**: Consumption of pro-inflammatory diets increases colorectal cancer risk among Japanese men; alcohol consumption further increases this risk for drinkers. These findings suggest that colorectal cancer may be prevented through dietary modification.

## 1. Introduction

Colorectal cancer is the third most common malignancy globally, with an estimated 1.9 million new cases and 930,000 deaths in 2020 [1]. Although genetic factors contribute to its development, most cases are considered sporadic and largely related to modifiable lifestyle factors characteristic of Western culture, including unhealthy diets, obesity, physical inactivity, alcohol consumption, and smoking [2].

Inflammation is a key mechanisms underlying the pathogenesis of colorectal cancer [2]. Diet is a strong mediator of inflammation, and the relationship between diet and inflammatory conditions is complex: some nutrients and foods increase inflammation, whereas others decrease it. For example, an animal-derived nutrient pattern—characterized by high intake of animal protein, cobalamin, cholesterol, and omega-6 fatty acids—was positively associated with C-reactive protein (CRP) levels, whereas a plant-derived nutrient pattern—characterized by high intake of beta-carotene, vitamin A, lutein, and zeaxanthin—was inversely associated with CRP levels [3]. A previous study reported that processed meat consumption was associated with higher CRP levels, whereas nut consumption was associated with lower CRP levels [4]. Additionally, a Japanese dietary pattern—characterized by high consumption of green tea, fish, and vegetables—has been reported to be negatively associated with CRP levels [5]. However, some studies have reported a positive association between fish consumption and inflammation [4]. This discrepancy may reflect the complex composition of the diet, highlighting the importance of assessing the inflammatory potential of the whole diet.

The dietary inflammatory index (DII) was developed to assess the inflammatory potential of diets [6,7]. Studies have shown that consumption of pro-inflammatory diets, as indicated by a high energy-adjusted-DII (E-DII^TM^) score, is a risk factor for the development of colorectal cancer [8]. Chronic inflammation levels are lower among Japanese people than among Western populations [9,10], possibly due to healthy eating habits. However, unhealthy lifestyles characterized by Westernization, including the consumption of pro-inflammatory diets, may increase the susceptibility of Japanese people to inflammatory responses. Although Japan has the longest life expectancy globally, the age-adjusted incidence rate of colorectal cancer is increasing over time [11].

The E-DII has been associated with a reduced risk of all-cause mortality in Japan, with no association observed for digestive cancer-related mortality [12]. Moreover, the E-DII may be linked to the development of colorectal cancer, as a high inflammatory status has been associated with early colorectal cancer incidence rather than invasive cancer [13]. Additionally, inflammation-inducing lifestyles such as alcohol consumption, smoking, physical inactivity, and obesity have been reported to increase colorectal cancer risk by 48–60% when combined with the consumption of pro-inflammatory diets [14]. Thus, a combination of unhealthy lifestyle habits can induce systemic inflammation, further increasing morbidity risk.

Although many studies examined the association between the E-DII and colorectal cancer, very few have been conducted in East Asia, and all were case–control studies. Therefore, this study aimed to (1) investigate the association between the E-DII score and colorectal cancer incidence in a prospective cohort study in Japan and (2) examine how different combinations of unhealthy lifestyle factors interact.

## 2. Materials and Methods

### 2.1. Study Participants

The Japan Public Health Center-based prospective study (JPHC Study) comprises two cohorts: Cohort I (1990) and Cohort II (1993). This study was conducted with the aim of gathering evidence to help maintain and improve health conditions, such as cancer. The JPHC Study, whose design has been described previously [15], included 68,722 male residents of 11 public health center areas across Japan who were aged 40–69 years at baseline. Follow-up surveys were conducted every 5 years. The 5-year follow-up surveys in 1995 for Cohort I and in 1998 for Cohort II were treated as the starting points of follow-up because the 5-year follow-up data included more comprehensive dietary information than the baseline survey. Participants were followed until 31 December 2013 (and until 31 December 2012 in the Osaka area only). The Tokyo area (*n* = 2919) was excluded because cancer incidence data were not available.

**Figure 1:**
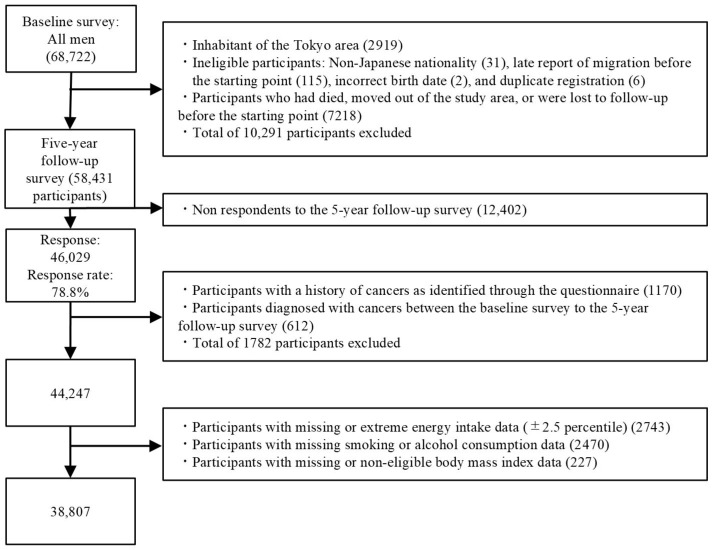
Flow chart of the participants in the Japan Public Health Center-based Prospective Study.

A total of 58,431 men were identified as eligible for follow-up after excluding participants who did not meet the inclusion criteria, died, moved out of the study area, or were lost to follow-up before the start of follow up (Figure 1). Of these, 46,029 men completed the 5-year follow-up (response rate, 78.8%). Participants were further excluded if they had a history of any cancer identified by the questionnaire (*n* = 1170) or were diagnosed between baseline and the 5-year follow-up (*n* = 612). Participants with missing or extreme energy intake data (upper and lower 2.5 percentiles) (*n* = 2743), missing smoking or alcohol consumption data (*n* = 2470), or missing or implausible body mass index (BMI) data (*n* = 227) were also excluded. Consequently, data from the remaining 38,807 men were included in the present study.

### 2.2. Dietary Assessment

At the 5-year follow-up, habitual dietary intake was assessed using a food frequency questionnaire (FFQ) consisting of 138 food and beverage items. For each food item, respondents were asked about the frequency of consumption and portion size per serving. Frequency of consumption was assessed using nine graded categories ranging from “less than once a month” to “≥7 times/day,” and portion size was divided into three response options: “less than half,” “standard,” and “more than 1.5 times.” For beverages, respondents were asked only about the frequency of consumption, with nine categories ranging from “<1 cup/week” to “≥10 cups/day.” Energy and nutrient intakes were calculated by multiplying the frequency of food intake by portion size and nutrient content per 100 g. Nutrient content data were obtained from the Fifth Revised and Enlarged Edition of the Standard Tables of Food Composition in Japan [16].

E-DII scores were calculated using intake data for 30 nutrients and foods obtained from the FFQ [6,7]. Briefly, the E-DII was developed through an extensive review of the scientific literature that identified 45 nutrients and foods associated with inflammation as components of the index. This review included 1943 articles (cell culture, animal, and human studies). The articles were weighted according to study design and number to determine the pro- or anti-inflammatory effects of each E-DII component. Individual dietary intake data, standardized to the global daily mean intake, were then multiplied by their respective inflammatory effect scores and summed to calculate an participant’s total E-DII score. A positive score indicates a pro-inflammatory diet, whereas a negative score indicates an anti-inflammatory effect. In the present study, 30 of the 45 components were used because intake data could not be calculated for some components or because their intakes are very low in the Japanese population (details of the E-DII components are listed in Table S1). This approach is well established in E-DII research, and previous studies have shown that the association between the DII score and CRP levels remain robust even when the number of dietary components is reduced from 44 to 28 [7]. To compute the E-DII scores, intakes were standardized per 1000 kcal of energy intake, and z-scores were calculated based on the energy-adjusted global comparative database. These z-scores were then converted to proportions that were centered on 0 by doubling and subtracting 1. Individual food parameter scores were then summed to create the overall E-DII score.

A validation study of the DII conducted in a subset of the study participants [9] showed that the correlation (r) between the FFQ and dietary record, used as the reference method, were 0.35 in Cohort I and 0.48 in Cohort II. These correlation coefficients are considered acceptable in nutritional epidemiology for ranking individuals by habitual intake and are consistent with median values reported in Japanese (0.31–0.56) and international (0.37–0.56) FFQ validation reviews [17,18]. Furthermore, a positive trend was observed for interleukin (IL)-6, an inflammatory marker. The geometric means IL-6 concentrations in the lowest and highest E-DII quartiles were 1.14 and 1.33 pg/mL (*p* for trend = 0.05) among men in Cohort I, and 1.07 and 1.14 pg/mL (*p* for trend = 0.42) among men in Cohort II, respectively. Because no positive trends in inflammatory markers were observed among Japanese women, only men were included in this study.

### 2.3. Identification of Colorectal Cancers

Colorectal cancer incident cases were identified by population-based cancer registries and hospital records in the study areas. Death certificate files were also used as supplemental data. The International Classification of Diseases for Oncology, Third Revision was used to code colorectal cancer (C18–C20). In addition, colorectal cancer was divided into groups by site: proximal colon (C18.0–C18.5), distal colon (C18.6 and C18.7), and rectum (C19 and C20). Proximal, distal, overlapping sites (C18.8), and unspecified sites (C18.9) were combined and defined as colon cancer. The proportion of death certificate notifications was 3.7%, and that of death certificate only (DCO) was 2.5%. Additionally, 96% of cases were pathologically confirmed. In accordance with international standards for cancer registration quality (e.g., IARC/IACR guidelines), a DCO proportion below 10% is recognized as indicative of high completeness and accuracy [19].

### 2.4. Statistical Analysis

Means, standard deviations (SDs), and proportions were calculated according to quintiles of E-DII scores. To compare characteristics between quintiles, *p*-values were calculated using the Jonckheere–Terpstra test for continuous variables and the chi-squared test for categorical variables. Person-years of follow-up were calculated from the date of response to the 5-year follow-up survey to the date of colorectal cancer diagnosis, death, migration out of the study area, or end of follow-up, whichever occurred first. For participants lost to follow-up, the last confirmed date of presence in the study area was used as the censoring date.

Hazard ratio (HRs) and 95% confidence intervals (CIs) were calculated to assess colorectal cancer across DII score quintiles using Cox proportional hazards regression models, with the lowest category as the reference. By assigning ordinal values to E-DII score quintiles, trends in HRs were also assessed. HRs were adjusted for the following potential confounders: age (years), area (10 areas with public health center), smoking status (current, past, and never), BMI (kg/m^2^; <22.5, 22.5–24.9, 25–27.4, and ≥27.5), total physical activity (metabolic equivalent of tasks, hours per day, continuous), log-transformed total energy intake (continuous), energy-adjusted red and processed meat intake (continuous), energy-adjusted calcium intake (continuous), history of diabetes (yes or no), and family history of colorectal cancer (yes or no). These covariates were selected *a priori* based on established evidence from WCRF/AICR reports [20] and previous cohort studies, with careful consideration of causal pathways to distinguish confounders from intermediate variables, thereby avoiding overadjustment for components of the E-DII or closely related factors (such as vegetables and fruit). Additional analyses further adjusting for dietary supplement use indicated that the results were materially unchanged; therefore, dietary supplement use was not retained in the final multivariable models. Alcohol intake was not included as a potential confounder in the main multivariable models because it is also a component of the E-DII score. Instead, alcohol consumption was assessed as an effect modifier through stratified analyses. For sensitivity analyses, participants with cancer incidence during the first 3 years of follow-up were excluded.

Stratified analyses were performed to evaluate whether the association between the E-DII score and colorectal cancer risk was affected by modifiable potential lifestyle risk factors: BMI status (<25 and ≥25 kg/m^2^), smoking status (non-smoker and current/past-smoker), alcohol drinking status (non-drinker and drinker), and level of physical activity (median number of MET-hours, <31.85 and ≥31.85). To assess statistical interactions, an interaction term was created by multiplying E-DII score quintiles by each dichotomous lifestyle variables and added to the model. When a significant interaction was observed, additional adjustment for the stratifying factor within strata was conducted as a sensitivity analysis to assess potential residual confounding. Two-sided *p*-values < 0.05 were considered statistically significant, and all statistical analyses were performed using SAS (version 9.4; SAS Institute Inc., Cary, NC, USA).

## 3. Results

### 3.1. Characteristics of the Participants

As shown in Table 1, mean ± SD E-DII scores were −0.32 ± 1.87 overall, −3.1 ± 0.9 in the lowest group (Q1), and 2.1 ± 0.7 in the highest group (Q5). Participants in the highest E-DII quintile (Q5) were younger (mean age: 54.8 years in Q5 versus [vs.] 58.2 years in Q1), more likely to be current smokers (53.4% vs. 37.4%), and had lower levels of physical activity (32.0 MET h/day vs. 32.7 MET h/day), as well as a lower prevalence of diabetes (7.7% vs. 12.0%) than those in the lowest quintile (Q1). Regarding dietary factors, individuals in Q5 had lower energy intake (2031 kcal/day vs. 2265 kcal/day) and calcium intake (397 mg/day vs. 609 mg/day), and higher intakes of red and processed meat (52.1 g/day vs. 48.9 g/day) and alcohol (268 g/week vs. 139 g/week) than those in Q1. The distributions of E-DII components and food group intakes are presented in Tables S1 and S2.

### 3.2. Association Between the E-DII and Colorectal Cancers

As shown in Table 2, a total of 1415 cases of colorectal cancer were diagnosed during a mean follow-up of 14.1 years, yielding a crude incidence rate of 259.1 per 100,000 person-years. This rate is comparable with the national age-specific incidence reported for Japanese men in 2005 (the midpoint of the study period), with rates of 191.5, 266.7, and 350.5 per 100,000 among those aged 60–64, 65–69, and 70–74 years, respectively [21,22].

In a model adjusted for age and area, colorectal cancer risk significantly increased among participants who consumed pro-inflammatory diets (*p* for trend = 0.006; the HR [95% CI] at Q5 was 1.28 [1.08–1.53]). In the multivariable-adjusted model, consumption of pro-inflammatory diet was also associated with an increased risk of colorectal cancer, although the association was slightly weaker (*p* for trend = 0.08; the HR [95% CI] at Q5 was 1.20 [0.99–1.46]). In sensitivity analysis excluding individuals diagnosed within the first 3 years of follow-up, the positive association between the E-DII score and colorectal cancer incidence was stronger (*p* for trend = 0.04; the HR [95% CI] at Q5 was 1.28 [1.04–1.57]).

### 3.3. Stratified Analysis

Stratified analysis by colorectal cancer site revealed differences in the effect of the E-DII score on cancer incidence. Consumption of pro-inflammatory diet was significantly associated with an increased risk of colon cancer incidence in the multivariate-adjusted model (*p* for trend = 0.03; the HR [95% CI] at Q5 was 1.28 [1.01–1.63]). In contrast, there was no significant association between the E-DII score and the risk of rectal cancer (*p* for trend = 0.99; the HR [95% CI] at Q5 was 1.06 [0.75–1.48]). These associations remained consistent in a sensitivity analysis excluding cases within the first 3 years of follow-up.

Furthermore, when colon cancer was stratified into proximal and distal colon cancers, a significant association was observed between pro-inflammatory diet consumption and proximal colon cancer risk, whereas no significant association was found for the distal colon cancer risk (proximal colon cancer, *p* for trend = 0.045, HR at Q5 [95% CI]: 1.32 [0.92–1.90]; distal colon cancer, *p* for trend = 0.35, HR [95% CI] at Q5: 1.20 [0.87–1.67]). However, the point estimates of the HRs were comparable for proximal and distal colon cancers.

Table 3 shows the results of the analysis of interactions between inflammation-inducing lifestyle factors and a one-unit change in E-DII score quintiles. Among less physically active participants, colon and proximal colon cancer risks increased with the consumption of pro-inflammatory diets, but no significant interaction was observed. Similar findings were observed for BMI and smoking status. However, stratified analysis of drinking habits showed no significant association among non-drinkers, but there were positive associations between the E-DII and incidences of colorectal, colon, and proximal colon cancers among drinkers, with a significant interaction for proximal colon cancer (*p* for interaction = 0.01). After adjusting for alcohol intake, a significant positive association remained between the E-DII score and proximal colon cancer among drinkers (one-unit change HR [95% CI]: 1.13 [1.03–1.24]). The HRs for E-DII score quintiles according to lifestyle factors are outlined in Table S3.

## 4. Discussion

The results of the present study indicate that consumption of a pro-inflammatory diet is associated with an increased risk of colorectal cancer, particularly colon cancer. However, when interactions with lifestyle habits related to chronic inflammation were examined, no significant interactions were observed for BMI, smoking, or physical activity. The only significant interaction identified was for drinking habits. Consumption of a pro-inflammatory diet increased the risk of proximal colon cancer among alcohol drinkers, compared with non-drinkers.

This is the first prospective study to examine the association between the consumption of a pro-inflammatory diet and colorectal cancer incidence among Japanese men. A meta-analysis of the association between the E-DII and colorectal cancer [23] showed that a high E-DII was associated with an increased risk of colorectal cancer among men (highest vs. lowest risk ratio [RR] [95% CI] = 1.51 [1.29–1.76]). However, when stratified by study design, the RR [95% CI] was 1.27 [1.16–1.38] for prospective studies, suggesting that the inclusion of case–control studies in the meta-analysis may have led to an overestimation of the risk. The HR in our study was comparable to that of the prospective studies in the meta-analysis. Additionally, all the prospective studies included in the meta-analysis were conducted in Western countries. Considering that this is the first demonstration of an association between the E-DII and colorectal cancer risk among Japanese men, the results suggest that consumption of a pro-inflammatory diet, as assessed by the E-DII, may increase colorectal cancer risk in a diverse population. As Japan is now regarded as a high-incidence country for colorectal cancer [24,25], the present findings may be informative for other high-risk populations internationally.

In the anatomic site-specific analysis of the present study, consumption of a pro-inflammatory diet was associated with a significantly increased risk of colon cancer. A meta-analysis of prospective studies examining the association between the E-DII and site-specific colorectal cancer [23] also revealed a significant positive association for colon cancer (RR [95% CI] = 1.20 [1.11–1.30]), whereas no association was observed for rectal cancer (RR [95% CI] = 1.07 [0.87–1.31). Similar site-specific disparities have been reported in previous studies focusing on blood levels of CRP [13,26]. These observations, together with our findings, suggest differing susceptibilities to inflammation between the colon and rectum. Among patients with ulcerative colitis, those whose inflammation is primarily affecting the colon have an increased risk of colon cancer, whereas those whose inflammation is limited to the rectum do not have an increased risk of rectal cancer [27]. Additionally, for some nutrients that comprise the E-DII, such as vitamin C [28], folic acid [29], and magnesium [30,31], low intakes have been associated with an increased risk of colon cancer but not rectal cancer. Thus, the results of the present study may represent a combined effect of several nutrients. Further studies are required to determine whether the inflammation-modifying effects of diet differentially influence site-specific cancer incidence.

No significant interactions were found between E-DII and BMI, smoking, or physical activity. In previous studies, stratified analysis revealed an increased risk of colorectal cancer, especially in groups with unhealthy lifestyles, but interaction results have been inconsistent [32,33]. In contrast, a significant interaction with drinking habits was observed in the present study, with an increased risk of proximal colon cancer observed among drinkers. Furthermore, association remained positive in drinkers, even after adjusting for alcohol intake. Alcohol is included as an anti-inflammatory component of the E-DII (inflammation coefficient = −0.278) [6], based on scientific literature available at the time of its development [34,35]. More recent evaluations, including those by the World Health Organization, emphasize that alcohol consumption, even at low levels, carries health risks [36]. In this context, the observed positive association between E-DII scores and alcohol intake in our study may reflect typical dietary habits among alcohol drinkers, such as lower vegetable intake and higher consumption of red and processed meats. Similar results have been reported previously [32], suggesting that consumption of a pro-inflammatory diet may be a characteristic dietary pattern among alcohol drinkers.

Alcohol is a well-established risk factor for colorectal cancer [37]; thus, a stratified analysis was performed in this study. Although many previous studies have eliminated the possibility of confounding by including alcohol in their models [33,38], few studies have been conducted to examine interactions with drinking habits, especially the risk of pro-inflammatory diets in drinkers. Only one study has examined the potential interaction [33]; however, no interaction was found. Nevertheless, similar to the present study, that study reported a positive association between the E-DII and colorectal cancer among heavy drinkers and a null association among non-drinkers. Alcohol disrupts the intestinal epithelial barrier, leading to mucosal immunity, intestinal inflammation, and even systemic inflammation [39]. Moreover, alcohol affects nutrient metabolism, and low intakes of micronutrients such as folic acid [29,40] and magnesium [30] have been reported to increase the risk of colorectal cancer among alcohol drinkers. Although the possibility of residual confounding by alcohol should be noted, the E-DII may have further increased the risk of colorectal cancer among drinkers. Further research is warranted to clarify the biological mechanisms linking alcohol consumption, diet-related inflammation, and colorectal cancer risk.

Although the relationship between the E-DII and colorectal cancer has been widely investigated, data from East Asia are scarce [41], and all available studies to date have employed case–control methodologies. The JPHC Study is a large prospective cohort study of lifestyle-related diseases in which exposure information was collected before the cancer was diagnosed, thereby minimizing recall bias. Participants were selected from the general population, and the response rate for the 5-year follow-up survey was approximately 80%. The loss to follow-up rate was quite low, with fewer than 10% of DCOs. Thus, the follow-up data and cancer registry information for this study population were of adequate quality. In a study of the association between the E-DII and risk of death among Japanese populations, no significant association was observed with digestive cancer [12]. The present findings, derived from a population with a low chronic inflammatory status, provide important evidence supporting the potential for disease prevention through dietary modification, a modifiable lifestyle habit, across diverse populations.

Despite its strengths, this study has some limitations. Some degree of selection bias cannot be ruled out. In this cohort, non-respondents to the 5-year follow-up survey were slightly younger than respondents (54.6 ± 7.5 years vs. 56.8 ± 7.9 years), and previous studies have reported higher mortality among non-respondents than among respondents [42,43]. If such differences also extend to dietary inflammatory potential, the exclusion of non-respondents may have resulted in an underestimation of the observed association between the E-DII and colorectal cancer risk in this study. The FFQ is a relative dietary assessment method, and misclassification may have attenuated the observed associations. Although, the validity of E-DII assessment by the FFQ has been previously investigated using dietary records and inflammatory biomarkers, particularly among Japanese men, some degree of measurement error is inevitable and may have led to an underestimation of the true association. Additionally, we were unable to account for dietary changes during the follow-up period. Colorectal cancer risk has been reported to increase among individuals whose E-DII shifts toward a more pro-inflammatory profile during follow-up, as well as among those consuming pro-inflammatory diets compared with those consuming anti-inflammatory diets [44]. Therefore, the lack of consideration for dietary changes may have further attenuated the observed association. Future studies incorporating repeated dietary assessments are needed to evaluate the impact of changes in dietary inflammatory potential on colorectal cancer risk. The possibility of residual confounding from unmeasured factors, including a history of inflammatory bowel disease, also cannot be ruled out in the present study. Lastly, we did not examine sex differences because the validity of the E-DII derived from the FFQ has not been established in Japanese women. In our previous validation study, the E-DII was positively associated with inflammatory biomarkers in men, whereas no such association was observed in women. Furthermore, preliminary analyses in the same cohort showed no positive association between the E-DII and colorectal cancer risk among women. As it remains unclear whether these null findings reflect exposure misclassification or a true absence of association, we excluded women from the present analysis to avoid potential misinterpretation.

## 5. Conclusions

These findings suggest the potential for colorectal cancer prevention through dietary modification. Site-specific analyses revealed differential associations between colon and rectal cancer, highlighting the need for further investigations into the mechanisms underlying site-specific susceptibility to diet-related inflammation. However, as the validity of the FFQ-derived E-DII has not been established in Japanese women, the findings of this study may not be generalizable to women.

## Figures and Tables

**Table 1:** Characteristics of the study participants by E-DII quintiles in the Japan Public Health Center-based prospective study.

	E-DII Score										
	Q1 (Lowest)		Q2		Q3		Q4		Q5 (Highest)		*p*-Value ^a^
Number of participants	7761		7762		7761		7762		7761		
DII score, mean and SD ^b^	−3.1	(0.9)	−1.2	(0.4)	−0.2	(0.3)	−0.2	(0.3)	2.1	(0.7)	
Age at the 5 y follow-up survey, y	58.2	(7.6)	57.0	(7.6)	56.2	(7.5)	55.2	(7.4)	54.8	(7.6)	<0.0001
Height, cm	163.6	(6.4)	164.0	(6.2)	164.2	(6.3)	164.4	(6.3)	164.0	(6.4)	<0.0001
Body mass index at the 5 y follow-up survey, kg/m^2^	23.7	(2.8)	23.5	(2.8)	23.6	(2.8)	23.5	(2.9)	23.6	(3.0)	0.0002
Smoking status, %											<0.0001
Current	37.4		43.7		48.1		50.8		53.4		
Past	20.1		19.6		17.9		17.3		15.5		
Never	42.5		36.6		34.1		31.9		31.1		
Diabetes, %	12.0		10.0		8.7		7.5		7.7		<0.0001
Family history of colorectal cancer, %	1.4		1.4		1.3		1.2		1.1		0.59
Total physical activity, MET h/day	32.7	(6.9)	32.7	(7.0)	32.5	(6.9)	32.5	(7.0)	32.0	(7.1)	<0.0001
Dietary intake											
Total energy intake, kcal/day	2265	(650)	2229	(639)	2183	(627)	2124	(617)	2031	(639)	<0.0001
Red and processed meat, g/day	48.9	(31.9)	52.7	(34.2)	52.7	(34.7)	53.0	(37.1)	52.1	(44.7)	0.002
Calcium, mg/day	609	(182)	528	(184)	428	(187)	440	(209)	397	(258)	<0.0001
Alcohol, g/week	139	(166)	182	(195)	211	(218)	239	(251)	268	(320)	<0.0001

E-DII, energy-adjusted dietary inflammatory index; MET, metabolic equivalent of task; SD, standard deviation; Q, quintile. Data are shown as mean (SD) or percentage. The distributions for E-DII components and related food groups are presented in Tables S1 and S2. ^a^ The Jonckheere–Terpstra test was performed for continuous variables, and the chi-squared test was performed for categorical variables. ^b^ The E-DII score was calculated using dietary intakes standardized per 1000 kcal of energy intake.

**Table 2:** HRs and 95% CIs for colorectal cancer risk according to the E-DII quintiles in the Japan Public Health Center-based prospective study.

		E-DII Score									
		Q1 (Lowest)	Q2		Q3		Q4		Q5 (Highest)		*p* for Trend
		HR (Ref.)	HR (95% CI)		HR (95% CI)		HR (95% CI)		HR (95% CI)		
	Total	*n* = 7761	*n* = 7762		*n* = 7762		*n* = 7762		*n* = 7762		
**Colorectal cancer**											
Total person-y	546,190	108,822	109,237		109,693		109,981		108,458		
Number of cases	1415	257	302		298		288		270		
Age- and area-adjusted		1.00	1.24	(1.05–1.46)	1.26	(1.06–1.49)	1.29	(1.08–1.52)	1.28	(1.08–1.53)	0.006
Multivariable-adjusted ^a^		1.00	1.22	(1.03–1.45)	1.22	(1.03–1.45)	1.24	(1.04–1.49)	1.20	(0.99–1.46)	0.08
Multivariable-adjusted ^a^ (excluding cases <3 y)		1.00	1.31	(1.09–1.57)	1.25	(1.04–1.50)	1.32	(1.09–1.60)	1.28	(1.04–1.57)	0.04
**Colon cancer**											
Total person-y	541,879	108,047	108,282		108,834		109,176		107,541		
Number of cases	957	171	203		202		200		181		
Age- and area-adjusted		1.00	1.26	(1.03–1.54)	1.29	(1.05–1.58)	1.36	(1.10–1.67)	1.32	(1.07–1.63)	0.008
Multivariable-adjusted ^a^		1.00	1.25	(1.02–1.54)	1.28	(1.04–1.58)	1.35	(1.09–1.67)	1.28	(1.01–1.63)	0.03
Multivariable-adjusted ^a^ (excluding cases < 3 y)		1.00	1.35	(1.08–1.68)	1.34	(1.07–1.68)	1.44	(1.14–1.82)	1.37	(1.07–1.77)	0.02
**Proximal colon cancer**											
Total person-y	536,755	107,202	107,202		107,723		108,152		106,476		
Number of cases	404	74	87		86		90		67		
Age- and area-adjusted		1.00	1.26	(0.92–1.71)	1.30	(0.95–1.78)	1.46	(1.07–1.99)	1.20	(0.85–1.68)	0.14
Multivariable-adjusted ^a^		1.00	1.31	(0.96–1.79)	1.37	(1.00–1.89)	1.60	(1.15–2.21)	1.32	(0.92–1.90)	0.045
Multivariable-adjusted ^a^ (excluding cases < 3 y)		1.00	1.49	(1.06–2.08)	1.58	(1.12–2.23)	1.72	(1.21–2.46)	1.35	(0.91–2.02)	0.06
**Distal colon cancer**											
Total person-y	537,428	107,297	107,299		107,802		108,194		106,836		
Number of cases	507	87	106		106		104		104		
Age- and area-adjusted		1.00	1.28	(0.96–1.70)	1.30	(0.98–1.73)	1.34	(1.01–1.79)	1.42	(1.06–1.89)	0.02
Multivariable-adjusted ^a^		1.00	1.22	(0.92–1.63)	1.21	(0.90–1.62)	1.21	(0.89–1.64)	1.20	(0.87–1.67)	0.35
Multivariable-adjusted ^a^ (excluding cases < 3 y)		1.00	1.28	(0.94–1.74)	1.17	(0.85–1.61)	1.30	(0.94–1.80)	1.34	(0.94–1.89)	0.15
**Rectal cancer**											
Total person-y	537,030	107,309	107,267		107,660		108,025		106,769		
Number of cases	458	86	99		96		88		89		
Age- and area-adjusted		1.00	1.21	(0.90–1.61)	1.20	(0.89–1.60)	1.15	(0.85–1.55)	1.22	(0.90–1.66)	0.31
Multivariable-adjusted ^a^		1.00	1.17	(0.87–1.57)	1.12	(0.83–1.52)	1.05	(0.77–1.45)	1.06	(0.75–1.48)	0.99
Multivariable-adjusted ^a^ (excluding cases < 3 y)		1.00	1.24	(0.91–1.69)	1.08	(0.78–1.49)	1.11	(0.79–1.56)	1.11	(0.77–1.59)	0.86

HR, hazard ratio; CI, confidence interval; E-DII, energy-adjusted dietary inflammatory index; Q, quintile. ^a^ The multivariable-adjusted model was stratified by the public health center area (10 areas) and adjusted for age at the 5 y follow-up survey (y, continuous), smoking status (current, past, and never), body mass index (<22.5, 22.5–24.9, 25–27.4, and ≥27.5), total physical activity (METs, continuous), log-transformed total energy intake (continuous), energy-adjusted red and processed meat intake (continuous), history of diabetes (yes or no), family history of colorectal cancer (yes or no), and energy-adjusted calcium intake (continuous).

**Table 3:** HRs and 95% CIs for colorectal cancer risk per one-unit change in E-DII quintiles, stratified by inflammation-related lifestyle factors, with interaction analyses.

		Total	Cases	One-Unit Change of E-DII Quintiles HR (95% CI)		*p* for Interaction
**Body mass index**						
Colorectal cancer	≤25 kg/m^2^	27,752	996	1.04	(0.99–1.09)	0.91
	>25 kg/m^2^	11,055	419	1.04	(0.97–1.12)	
Colon cancer	≤25 kg/m^2^		673	1.06	(1.00–1.12)	0.89
	>25 kg/m^2^		284	1.06	(0.98–1.16)	
Proximal colon cancer	≤25 kg/m^2^		271	1.05	(0.96–1.15)	0.25
	>25 kg/m^2^		133	1.15	(1.01–1.30)	
Distal colon cancer	≤25 kg/m^2^		362	1.05	(0.97–1.14)	0.44
	>25 kg/m^2^		145	1.00	(0.88–1.12)	
Rectal cancer	≤25 kg/m^2^		323	1.00	(0.92–1.09)	0.96
	>25 kg/m^2^		135	1.00	(0.88–1.13)	
**Smoking status**						
Colorectal cancer	Non-smokers	13,674	438	1.04	(0.97–1.12)	0.93
	Current or past smokers	25,133	977	1.04	(0.99–1.09)	
Colon cancer	Non-smokers		311	1.05	(0.97–1.14)	0.77
	Current or past smokers		646	1.06	(1.00–1.13)	
Proximal colon cancer	Non-smokers		144	1.10	(0.98–1.24)	0.70
	Current or past smokers		260	1.07	(0.97–1.18)	
Distal colon cancer	Non-smokers		151	1.03	(0.91–1.16)	0.90
	Current or past smokers		356	1.04	(0.95–1.13)	
Rectal cancer	Non-smokers		127	1.03	(0.91–1.17)	0.59
	Current or past smokers		331	0.99	(0.91–1.08)	
**Alcohol drinking status**						
Colorectal cancer	Non-drinkers	9305	271	0.98	(0.90–1.06)	0.07
	Drinkers	29,502	1144	1.06	(1.01–1.11)	
Colon cancer	Non-drinkers		195	1.00	(0.91–1.11)	0.19
	Drinkers		762	1.08	(1.02–1.14)	
Proximal colon cancer	Non-drinkers		94	0.93	(0.80–1.07)	0.01
	Drinkers		310	1.14	(1.05–1.25)	
Distal colon cancer	Non-drinkers		92	1.07	(0.93–1.24)	0.61
	Drinkers		415	1.03	(0.95–1.12)	
Rectal cancer	Non-drinkers		76	0.91	(0.78–1.07)	0.17
	Drinkers		382	1.03	(0.95–1.12)	
**Physical activity status**						
Colorectal cancer	<Median of METs	17,027	642	1.05	(0.99–1.11)	0.74
	≥Median of METs	21,780	773	1.03	(0.98–1.09)	
Colon cancer	<Median of METs		451	1.10	(1.03–1.19)	0.09
	≥Median of METs		506	1.02	(0.96–1.09)	
Proximal colon cancer	<Median of METs		203	1.13	(1.02–1.26)	0.24
	≥Median of METs		201	1.04	(0.94–1.16)	
Distal colon cancer	<Median of METs		225	1.08	(0.97–1.19)	0.28
	≥Median of METs		282	1.01	(0.92–1.10)	
Rectal cancer	<Median of METs		191	0.93	(0.83–1.03)	0.06
	≥Median of METs		267	1.05	(0.96–1.16)	

HR, hazard ratio; CI, confidence interval; E-DII, energy-adjusted dietary inflammatory index. The multivariable-adjusted model was stratified by the public health center area (10 areas) and adjusted for age at the 5 y follow-up survey (years, continuous), smoking status (current, past, and never), body mass index (<22.5, 22.5–24.9, 25–27.4, and ≥27.5), total physical activity (METs, continuous), log-transformed total energy intake (continuous), energy-adjusted red and processed meat intake (continuous), past history of diabetes (yes or no), family history of colorectal cancer (yes or no), and energy-adjusted calcium intake (continuous), excluding a factor using for the stratification.

## Data Availability

Individual data cannot be publicly shared due to participant privacy, in accordance with ethical guidelines in Japan. The informed consent obtained does not include permission for public data sharing. Qualifying researchers may apply to access a minimal dataset if they comply with certain requirements. Instructions are available at https://epi.ncc.go.jp/en/jphc/805/8155.html (accessed on 12 January 2026).

## Supplementary Materials

The following supporting information can be downloaded at https://www.mdpi.com/article/10.3390/nu18020338/s1. Table S1: Distributions of E-DII components by E-DII quintiles, JPHC Study, Japan; Table S2: Distributions of energy-adjusted food group intakes by E-DII quintiles, JPHC Study, Japan; Table S3: HRs and 95% CIs for colorectal cancer risk according to E-DII quintiles stratified by inflammation-related lifestyle factors.

## Abbreviations

The following abbreviations are used in this manuscript:

CRP	C-reactive protein
DII	Dietary inflammatory index
E-DII	Energy-adjusted-dietary inflammatory index
JPHC Study	Japan Public Health Center-based prospective study
FFQ	food frequency questionnaire
IL	interleukin
DCO	death certificate only
SDs	standard deviations
HRs	hazard ratios
CIs	confidence intervals
Q1	lowest group
Q5	highest group
RR	relative risk
